# A face-to-face comparison of claudin-5 transduced human brain endothelial (hCMEC/D3) cells with porcine brain endothelial cells as blood–brain barrier models for drug transport studies

**DOI:** 10.1186/s12987-020-00212-5

**Published:** 2020-08-26

**Authors:** Birthe Gericke, Kerstin Römermann, Andreas Noack, Sandra Noack, Jessica Kronenberg, Ingolf Ernst Blasig, Wolfgang Löscher

**Affiliations:** 1 Department of Pharmacology, Toxicology, and Pharmacy, University of Veterinary Medicine Hannover, Hannover, Germany; 2 Department of Trauma Surgery, Hannover Medical School, Hannover, Germany; 3 Leibniz-Institute for Molecular Pharmacology, FMP, Berlin, Germany; 4 Center for Systems Neuroscience, Hannover, Germany

**Keywords:** P-glycoprotein, Transwell, Porcine brain endothelial cells, Primary culture

## Abstract

**Background:**

Predictive in vitro models of the human blood–brain barrier (BBB) are essential in early drug discovery and development. Among available immortalized human brain capillary endothelial cell lines (BCECs), the hCMEC/D3 cell line has become the most widely used in vitro BBB model. However, monolayers of hCMEC/D3 cells form only moderately restrictive barriers, most likely because the major tight junction protein, claudin-5, is markedly downregulated. Thus, hCMEC/D3 monolayers cannot be used for vectorial drug transport experiments, which is a major disadvantage of this model.

**Methods:**

Here we transduced hCMEC/D3 cells with a claudin-5 plasmid and compared the characteristics of these cells with those of hCMEC/D3 wildtype cells and primary cultured porcine BCECs.

**Results:**

The claudin-5 transduced hCMEC/D3 exhibited expression levels (and junctional localization) of claudin-5 similar to those of primary cultured porcine BCECs. The transduced cells exhibited increased TEER values (211 Ω cm^2^) and reduced paracellular mannitol permeability (8.06%/h), indicating improved BBB properties; however, the barrier properties of porcine BCECs (TEER 1650 Ω cm^2^; mannitol permeability 3.95%/h) were not reached. Hence, vectorial transport of a selective P-glycoprotein substrate (*N*-desmethyl-loperamide) was not observed in claudin-5 transduced hCMEC/D3 (or wildtype) cells, whereas such drug transport occurred in porcine BCECs.

**Conclusions:**

The claudin-5 transduced hCMEC/D3 cells provide a tool to studying the contribution of claudin-5 to barrier tightness and how this can be further enhanced by additional transfections or other manipulations of this widely used in vitro model of the BBB.

## Background

The blood–brain barrier (BBB) protects the brain against numerous potentially toxic compounds, but also restricts passage of most medically used drugs [1–3]. The barrier function of the BBB is primarily a result of tight junctions (TJs) between adjacent brain capillary endothelial cells (BCECs) and high expression of active multidrug efflux transporters, including P-glycoprotein (Pgp; ABCB1; MDR1) and breast cancer resistance protein (BCRP; ABCG2), at the apical membrane of these cells [2–4]. A basal lamina, pericytes and astroglial endfeet, which envelop the endothelial cells, add to barrier function [2, 5]. As a consequence of the presence of TJs between BCECs, paracellular diffusion of polar compounds is almost impossible [2, 6, 7]. Small (< 400-600 Da) lipophilic and uncharged compounds can diffuse through the endothelial membrane, but their brain uptake is often restricted by active efflux transporters such as Pgp [2, 4]. Numerous in vitro models of the BBB, based on primary BCECs or immortalized BCEC lines, have been developed for studying drug transport [8]. Particularly for early drug discovery and development, reliable and screening-usable in vitro models of the human BBB are urgently needed [9]. However, current in vitro BBB models do not replicate the functions of the human BBB in vivo to sufficient extent [10]. Although several non-BCEC cell lines such as Caco-2 (a continuous line of heterogeneous human epithelial colorectal adenocarcinoma cells) or LLC-PK1 (Lilly Laboratories cells-porcine kidney 1) are widely used as surrogate models of the BBB to evaluate drug transport, they can obviously not replicate the complexity and predictive value of a true BBB-BCEC model [11]. Thus, validation of drug transport data using BCECs in more sophisticated models is usually necessary [11]. In this respect, primary cultures of bovine or porcine BCECs may be used, although the sequence of proteins expressed by these models differ from their human homologues, which may result in differences in drug affinities and transport rates [8]. Using human primary BCEC cultures would avoid such interspecies differences and pharmacogenomic variation, but the availability of such cells is ethically restricted [9]. Immortalized human BCECs can be used as an alternative [9].

Based on several recent reviews, the hCMEC/D3 cell line is the most widely used and most extensively characterized human BCEC line that is presently available for in vitro studies of the human BBB [8, 10, 12–14]. This cell line recapitulates a substantial number of BBB-BCEC characteristics, including the spindle-shaped morphology of BCECs and the expression and topographical distribution of several TJ proteins and BBB endothelial transporters and receptors, which renders hCMEC/D3 cells a reasonable approach for routine use. The only major limitation of these cells is that, due to a relatively low junctional tightness in monolayers, they cannot be used for bidirectional (vectorial) transport experiments of small molecules, whereas they seem to be well suited for mechanistic studies of BBB transporters and receptors [8].

The relatively low junctional tightness of hCMEC/D3 cells in monolayers as reflected by low transendothelial electrical resistance (TEER) and high paracellular permeability seems to be a consequence of the expression level of the major TJ protein claudin-5 (*encoded* by the *CLDN5* gene), which is important for junctional tightness, but is markedly lower in hCMEC/D3 than in intact microvessels [8]. This prompted us to transfect hCMEC/D3 cells with *Cldn5* and compare their BBB characteristics with those of wildtype (WT) cells. Furthermore, primary cultures of porcine BCECs (pBCECs) were used for comparison. The latter cells form tight endothelial monolayers with high TEER and low paracellular permeability and are ideally suited for investigations of small molecule transport across the BBB [8]. Our hypothesis was that *Cldn5* transfected hCMEC/D3 cells should exhibit similar barrier characteristics as pBCECs and thus provide a human BBB model suited for drug transport studies.

## Methods

### Generation of Cldn5-YFP-hCMEC/D3 cells

**Fig. 1:**
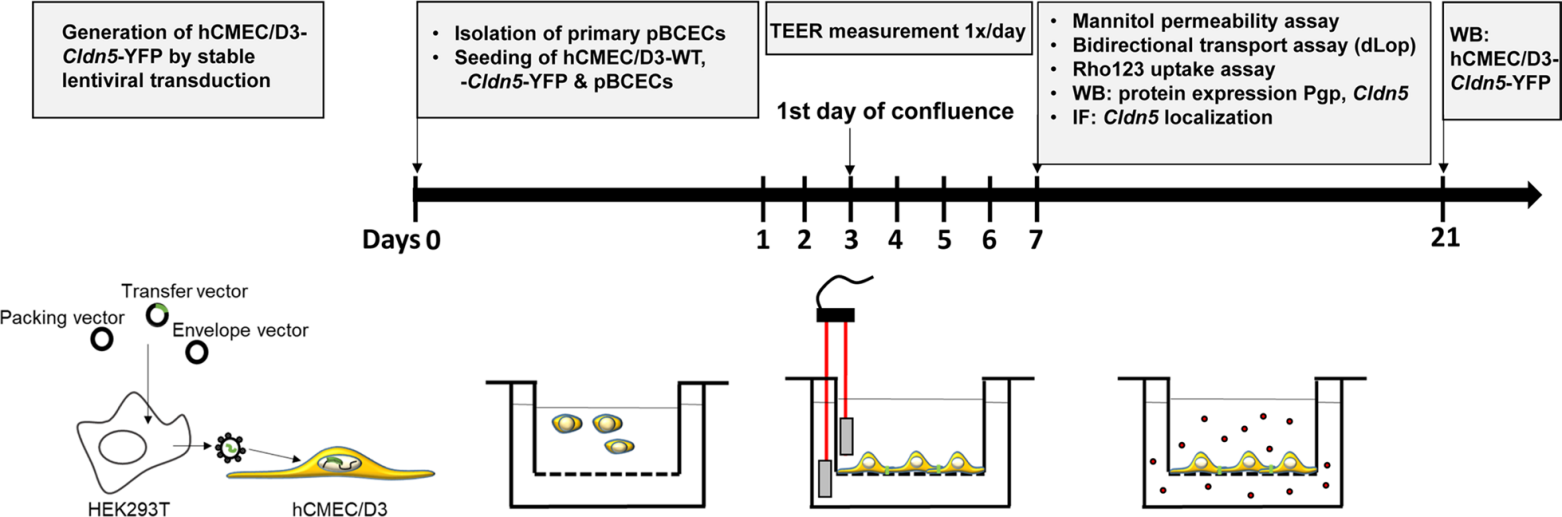
Schematic illustration of the experimental workflow. hCMEC/D3 cells stably expressing *Cldn5*-*YFP* were generated by lentiviral transduction. HEK293T cells were used as packing cell line to produce lentiviral particles for transduction of hCMEC/D3-WT cells. At day 0, prior to experiments, pBCECs were freshly isolated from porcine brain. On the same day, pBCECs, hCMEC/D3-WT and -*Cldn5*-YFP cells were seeded on semipermeable membranes of two chamber devices, each in triplicates. During experiments and cultivation, medium of hCMEC/D3-*Cldn5*-YFP cells was supplemented with doxycycline (1 µg/mL). After seeding cells attach, proliferate and differentiate to form a continuous monolayer with tight junctions sealing the paracellular cleft between adjacent cells. Transendothelial resistance (TEER) was measured once daily until 7 days after seeding of the cells, using an EVOM Volt-Ohm meter equipped with a STX2/chopstick, consisting of a fixed pair of electrodes. For comparison, TEER of hCMEC/D3 cells was also measured using an EndOhm-6 chamber (not illustrated). At day 7 after seeding, the cells were used for measuring paracellular permeability of mannitol and transcellular drug transport using the Pgp substrate *N*-desmethyl-loperamide (dLop). Pgp functionality was assessed by rhodamine 123 (Rho123) uptake assay. Another set of cells was grown to analyze *Cldn5* and Pgp expression by Western blot (WB) and Cldn5 localization by immunofluorescent staining (IF) 7 days after seeding. *Cldn5* expression in hCMEC/D3-*Cldn5*-YFP cells was also analyzed 21 days after seeding. Drawings are not to scale

The human brain endothelial cell line hCMEC/D3 [15] was kindly provided by Dr. Pierre-Olivier Couraud (Institute COCHIN, Paris, France). This hTERT/SV40-immortalized clonal cell line is derived from human temporal lobe microvessels isolated from tissue resected during surgery for epilepsy [15]. The hCMEC/D3 cells preserve the in vivo endothelial phenotype at least until 35th passages, including the spindle-shaped morphology of BCECs and the expression and topographical distribution of several tight junction proteins and BCEC transporters and receptors [8, 10, 12]. For the present experiments, cells were used up to passage 33. In addition to WT cells, hCMEC/D3 cells were used for lentiviral transduction with a doxycycline-inducible murine *Cldn5*-yellow fluorescent protein (*Cldn5*-YFP) fusion plasmid (Fig. 1). For this purpose, the murine sequence of *Cldn5* was cloned into a multiple cloning site of pEYFP-N1-vector via Sal I and BamHI (BD Biosciences Clontech). C-terminally tagged *Cldn5*-YFP was applied as it revealed cell barrier properties (junctional localization, colocalization with occludin, and strand morphology, composition and functionality of TJs) comparable to N-terminally tagged TRQ-*Cldn5* or Flag-*Cldn5* in recent experiments in MDCK-II cells ([16, 17] and unpublished data). Doxycycline inducible *Cldn5*-YFP expressing hCMEC/D3 cells were generated via a lentiviral system (cf., Fig. 1) similar to the previous generation of *MDR1*-EGFP-hCMEC/D3 cells [18].

With respect to the use of murine *Cldn5* for transfection of hCMEC/D3 cells, alignments of human and mouse *Cldn5* are highly homologous (98.2%). Differences are mainly localized in transmembrane domain 3 and 4. However, molecular modelling studies show that the differences do not influence their helical structure of the domains and, hence, the claudin-5 function in the junction [19, 20]. Important for the barrier function of claudin-5 is its paracellular tightening activity, which is caused by its extracellular domain consisting of the two extracellular loops (ECLs) [21]. Both ECLs show 100% homology between mouse and human. That is why we do not expect any functional difference of mouse *Cldn5* transduced in human BCECs with respect of the barrier formation in this BBB model. As mouse and human claudin-5 are structurally the same, the integrity of the tight junction strand network cannot be changed in the host cell as reported earlier in similar experiments [22].

### Cell culture conditions for hCMEC/D3-Cldn5-YFP and hCMEC/D3-WT cells

Cells were maintained in 100 mm culture dishes coated with collagen type I (100 µg/mL). Cells were cultured in endothelial cell basal medium-2 (EBM-2, Lonza, Cologne, Germany) supplemented with 5% fetal calf serum (FCS, PAA Laboratories, Cölbe, Germany), 1% penicillin (100 U/mL), streptomycin (100 µg/mL) (Invitrogen, Karlsruhe, Germany), 5 µg/mL ascorbic acid (Sigma-Aldrich; Munich, Germany), 1% lipid concentrate (Invitrogen), 10 mM HEPES (Invitrogen) and 1 ng/mL basic FGF (Sigma-Aldrich). In addition, 1.4 µM hydrocortisone (Sigma-Aldrich) was included in the medium to reinforce BBB properties [8]. For induction of *Cldn5*-YFP expression in *Cldn5*-YFP-transduced hCMEC/D3 cells, 1 µg/mL doxycycline (Biochrom, Berlin, Germany) was added to the medium during cultivation and the experiments. For monitoring of transendothelial electrical resistance (TEER) cells were seeded in a Transwell^®^ system (6 well format, polyester membrane, 4.67 cm^2^ growth area, 0.4 µm pore size; Corning Costar Corporation, Cambridge, MA, USA, #3401) at a density of 5 × 10^4^ cells/cm^2^.

### Primary cultures of porcine brain capillary endothelial cells

Primary porcine brain capillary endothelial cells (pBCECs) were isolated as described earlier [23], with slight modifications. In brief, fresh porcine brain hemispheres from *Sus scrofa domestica* (domestic pig) were kindly provided by the local slaughterhouse in Hannover (Germany) and stored on ice for transport. First, meninges and large blood vessels were removed from the cerebral cortex under sterile conditions. After roughly separating white from gray matter, the gray matter was minced and sequentially treated with digestion enzymes, density gradient centrifugation, filtration and erythrocyte lysing as described earlier [19]. Purified pBCECs were seeded in the apical compartment of collagen IV-coated Transwell^®^ chambers (12 well format, 0.4 µm pore size, 1.12 cm^2^ growth area, polyester membrane, transparent, Corning Costar, #3460) at 37 °C and 5% CO_2_ in a density of 3.6 × 10^5^ cells/cm^2^ in medium 199 (Gibco/Life Technologies, Carlsbad, CA, USA) supplemented with 10% newborn calf serum (Biochrom), 0.7 mM l-glutamine (Gibco/Life Technologies), 1% penicillin/streptomycin, and 1% gentamicin (Gibco/Life Technologies). Growth medium was exchanged to remove debris from attached cells after 1 h in culture. The next day 4 µg/mL puromycin (Sigma-Aldrich) was added to the cultures for the following 2 days to prevent growth of non-endothelial cells. For TEER measurement and a better differentiation of the cells growth, medium was exchanged to serum free assay medium composed of DMEM/F-12 with 0.7 mM glutamine, 100 U/mL penicillin, 100 mg/mL streptomycin, 100 mg/mL gentamicin and 550 nM hydrocortisone as described by Franke et al. [24].

For phase-contrast micrographs, pBCECs (or hCMEC/D3) were cultured in 100 mm culture dishes until 1–2 days post-confluency as described before. To assess cell morphology, the cell cultures were analyzed using an inverted fluorescence microscope (Olympus IX-70, Hamburg, Germany) with 10× magnification.

### Confocal fluorescence microscopy

Cells were seeded in the apical compartment of collagen-coated two-compartment chamber devices (translucent, PET membrane, 0.4 μm pore size, ThinCert, Greiner Bio-One, Frickenhausen, Germany, #657640) and fixed with 4% paraformaldehyde in PBS for 30 min at RT, followed by permeabilization with 0.2% Triton X-100 in PBS for 30 min at RT. Blocking and incubation of the cells with Alexa Fluor 568 phalloidin (#A12380, Thermo Fisher Scientific, Bonn, Germany; 1:100) for visualization of actin was performed in PBS containing 1% BSA, 0.5% saponin and 0.1% Triton X-100. An indirect staining of claudin-5 in hCMEC/D3-WT and primary pBCECs was conducted by using a primary claudin-5 antibody (#34-1600, Thermo Fisher Scientific, 1:100) and a secondary Alexa Fluor 488 antibody (#A11001, Thermo Fisher Scientific, 1:500). The membranes were cut out of the inserts and mounted on glass slides in Prolong Gold antifade reagent with DAPI (Carl Roth, Karlsruhe, Germany). Finally cover slips were placed upon the membranes. Samples were examined by a Leica TCS SP5 II confocal microscope (Leica Microsystems, Bensheim, Germany) with a HCXPL APO 63x lambda blue 1.4 oil immersion objective. Excitation wavelengths of 488 nm (*Cldn5*-YFP), 405 nm (DAPI) and 530 nm (Alexa Fluor 568 phalloidin) were used. Emission ranges used were 495–533 nm (*Cldn5*-YFP), 412–480 nm (DAPI) and 570–630 nm (Alexa Fluor 568 phalloidin).

### Western blot analysis

The expression of claudin-5 in hCMEC/D3 cells or primary pBCECs was evaluated by immunoblot analysis. Cells were lysed in buffer containing 25 mM Tris–HCl pH 8, 50 mM NaCl, 0.5% (w/v) sodium deoxycholate (DOC) and 0.5% (w/v) Triton X-100 supplemented with complete protease inhibitors (Roche, Mannheim, Germany). Cell lysis was performed for 1 h at 4 °C after passing the whole cell extract through a 21 gauge needle repeatedly. Protein concentrations in the lysates were determined using a Pierce BCA Protein Assay Kit (Thermo Fisher Scientific) following the manufacturer’s instructions. Equal amounts of protein were separated by 10% or 12% SDS-PAGE and transferred onto a PVDF membrane by Western blotting. After blocking in 5% milk, the membrane was incubated with primary antibody, either anti-claudin-5 (#34-1600, Thermo Fisher Scientific, 1:400) or anti-actin (#A2066; Sigma, 1:5000) for 1 h at RT followed by a 3x wash with PBS-T (0.05% Tween 20). Pgp was detected using an anti-Pgp antibody (C219; #SIG-38710; Signet Laboratories, Dedham, MA, USA, 1:200). HRP-conjugated secondary antibodies used were anti-rabbit (#P0448, Dako, Hamburg, Germany, 1:1000) or anti-mouse (#P0260, Dako, Hamburg, Germany, 1:1000). Incubation with the secondary antibody was performed for 45 min at RT. Proteins were visualized by enhanced chemiluminescent peroxidase substrate (SuperSignal West Femto Chemiluminescent substrate, Thermo Fisher Scientific) and documented with a ChemiDoc XRS System and Quantity One software (Bio Rad, Munich, Germany).

### Measurement of TEER and paracellular permeability

TEER of the cell monolayers was measured by an EVOM Volt-Ohm resistance meter (World Precision Instruments, Berlin, Germany) equipped with STX2 chopstick electrodes (World Precision Instruments). For comparison with the chopstick electrodes, TEER values were also determined by using EndOhm-6 tissue resistance measurement chambers (World Precision Instruments) connected to the EVOM Volt-Ohm meter. A TEER of at least 100 Ohm cm^2^ is recommended in the FDA guidance for in vitro BBB drug transport studies [25]. The TEER reflects permeability of intercellular TJs for ions and was measured daily over a period of 7 days. Therefor hCMEC/D3 cells or primary pBCECs were seeded in the apical chamber of two-compartment devices. TEER values were obtained from 3 individual culture inserts per cell type and calculated relative to the surface area of the culture inserts (Ω cm^2^). The TEER values of coated Transwell inserts without cells were subtracted from TEER values obtained in the presence of cells. Paracellular permeability was determined by [^14^C]-mannitol. At day 7 after seeding (equal to day 5 post-confluency), [^14^C]-mannitol permeability of the hCMEC/D3-WT and *Cldn5*-YFP as well as pBCEC cell layer on the Transwell inserts was assessed in basolateral to apical direction (b-A). For this, cells were equilibrated in OptiMEM for 30 min at 37 °C and TEER values were routinely determined before and after the mannitol assay. Afterwards, OptiMEM containing [^14^C]-mannitol with a concentration of 33.4 KBq/mL was placed into the basolateral chamber, whereas the apical chamber contained OptiMEM without [^14^C]-mannitol. The plates were kept in a 37 °C incubator with 5% CO_2_ on a horizontal shaker at 55 rpm and samples were taken from the apical chamber after 30 min, 60 min, 120 min and 180 min. The concentration of radiolabeled mannitol in the samples was determined by liquid scintillation counting using a 1450 Microbeta Trilux counter (Perkin Elmer Wallac, Rodgau-Jügesheim, Germany). Finally, the apparent permeability (P_app_) was calculated according to Artursson [26] as described previously [27].

### Rhodamine 123 uptake assay

Uptake assays with the cell-permeant, cationic, green-fluorescent Pgp substrate rhodamine 123 (Rho123; Sigma-Aldrich) were performed to evaluate Pgp transport function in hCMEC/D3 cells as previously described [18]. In these assays, alterations in Pgp-mediated drug efflux are indirectly measured by determining intracellular concentrations of Pgp substrates such as Rho123. For the Rho123 uptake assay, cells were grown on collagen type I (100 µg/mL) (Invitrogen) coated 6-well plates (Greiner, Frickenhausen, Germany) for 7 days. The cells were incubated with 5 µM Rho123 (Sigma-Aldrich) in OptiMEM (Invitrogen) shaking for 2 h at 37 °C and 5% CO_2_. Cells were washed twice with PBS and scraped in 1 mL ice cold PBS and collected in 1.5 mL tubes, which were centrifuged 7 min at 130 g at 4 °C. The cell pellet was resuspended in 150 µL lysis buffer (25 mM Tris–HCl, 50 mM NaCl, 0.5% (w/v) DOC and 0.5% (w/v) Triton X-100). Fluorescence was measured with the FLUOstar OPTIMA (BMG Labtech, Ortenberg, Germany) and was calculated as absolute fluorescence in the cell lysate per mg of protein. To determine the function of Pgp, the Pgp inhibitor tariquidar (0.5 µM) was added to the cells 1 h before Rho123 and during the 2 h of incubation with Rho123. Furthermore, the assays were performed in the presence or absence of doxycycline (1 µg/mL) to determine whether doxycycline inhibited or modulated Pgp function. Cells were either treated or non-treated with doxycycline (1 µg/mL) for the culture period of 7 days. The experiment was performed twice and data of the two experiments were averaged for final analysis.

### Vectorial drug transport assays

For these experiments, we used a concentration equilibrium transport assay (CETA), which is more sensitive to identify Pgp substrates than assays with concentration gradient (vectorial) conditions [27]. In the CETA, the drug is added to both (apical and basolateral) sides of the monolayer, so that initial drug concentration is the same in both compartments, thereby minimizing passive diffusion across the cell monolayer. Particularly for lipophilic compounds, passive transcellular diffusion could form a bias in Transwell assays by concealing active transport, which we have previously demonstrated by comparing vectorial drug transport of various small lipophilic drugs in conventional bidirectional (concentration gradient) assays versus drug transport in CETA [27].

For the present CETA experiments, cells were seeded on collagen type I coated (hCMEC/D3) or collagen IV coated (pBCEC) two chamber devices (Transwell^®^). The assay was performed in triplicates 7 days after seeding and TEER values were measured before and after the experiment to evaluate the monolayer integrity. Cells were pre-incubated with OptiMEM in presence or absence of the Pgp inhibitor verapamil (50 µM) for 1 h at 37 °C in a humidified atmosphere (5% CO_2_). The volumes on the apical and basolateral sides were 1.5 mL and 2.5 mL, respectively. The pre-incubation medium was replaced by OptiMEM containing the radiolabeled Pgp substrate [^3^H]*N*-desmethyl-loperamide ([^3^H]dLop, 5 nM), a metabolite of loperamide, which was added to both sides of the cells (apical and basolateral chamber) at identical concentrations. For transport analysis, samples were taken in triplicates at 0, 30, 60, 120, 180 and 240 min. Concentrations of [^3^H]dLop in the samples were measured by liquid scintillation counting using a 1450 Microbeta Trilux counter (Perkin Elmer Wallac). The results of the individual transport assays are presented for each chamber as the percentage of the initial drug concentration vs. time.

### Statistics

Values are shown as mean ± standard error of the mean (SEM). Significant differences between groups were calculated by one-way or two-way analysis of variance (ANOVA), followed by Bonferroni or Tukey post hoc tests. Values were considered to be significantly different when *P *< 0.05. All analyses were performed with the GraphPad Prism 8.0 software (GraphPad Software Inc., La Jolla, CA, USA).

## Results

### Transduction of hCMEC/D3 cells with Cldn5-YFP leads to increased expression of claudin-5 between adjacent cells

**Fig. 2:**
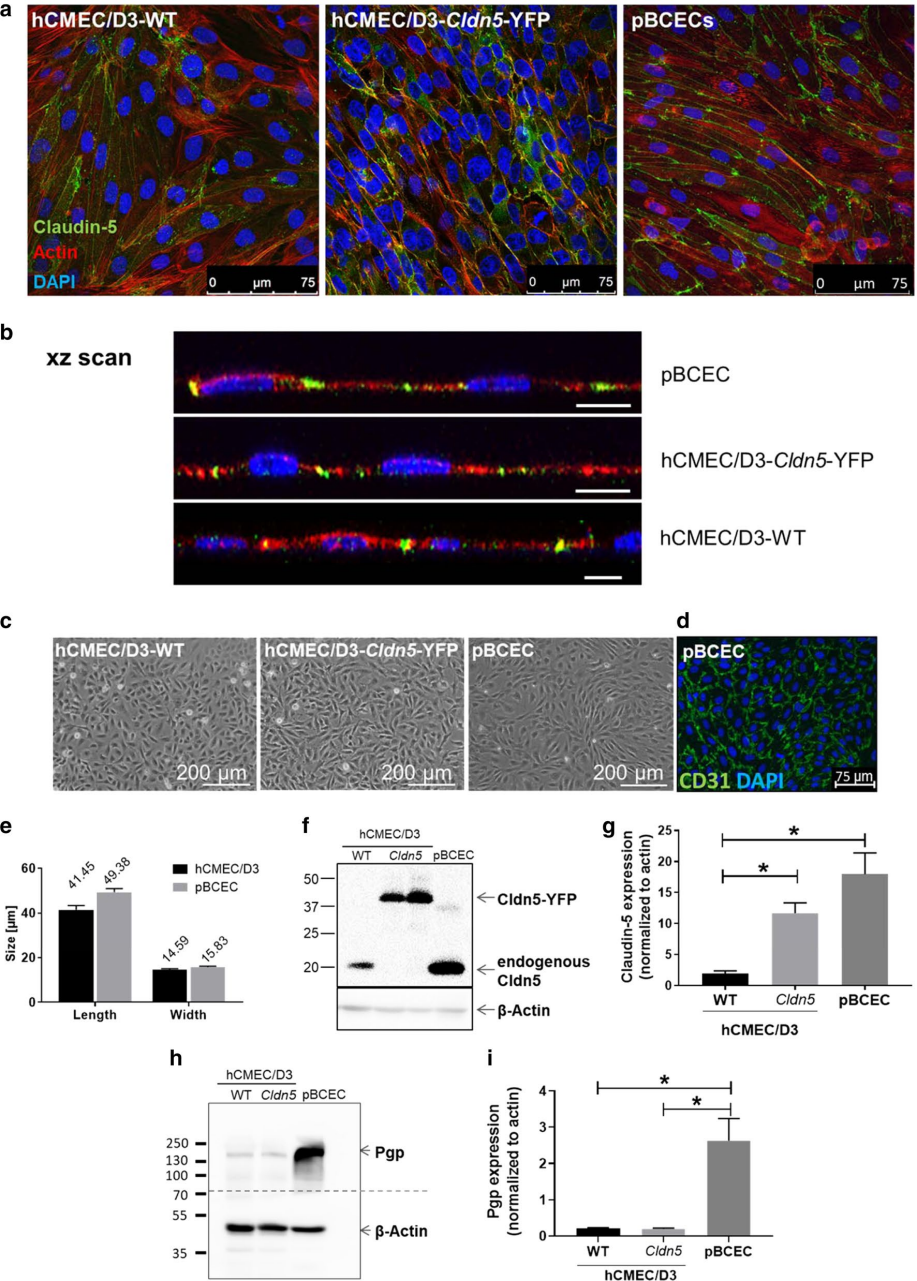
Comparison of cell morphology and localization and expression of claudin-5 and Pgp in hCMEC/D3-WT, hCMEC/D3-*Cldn5*-YFP and pBCECs. **a** Claudin-5 was indirectly stained (hCMEC/D3-WT and pBCECs) or visualized by YFP tag in the stably transduced hCMEC/D3-*Cldn5*-YFP cell line (for better visualization both claudin-5 and *Cldn5*-YFP are depicted in green). F-Actin is shown in red and cell nuclei are counterstained in blue by DAPI. **b** An xz scan of the cell layer revealed interendothelial (junctional) localization of claudin-5 (green) in the two cell lines and pBCECs. Scale bars: 10 µm. **c** Phase contrast micrographs of hCMEC/D3-WT, hCMEC/D3-*Cldn5*-YFP and primary pBCEC cultures. **d** Purity of pBCEC isolation, evaluated by fluorescent staining for the endothelial cell marker CD31 (green). Cell nuclei were counterstained with DAPI (blue). **e** Length and width of hCMEC/D3 and pBCECs. **f** Representative Western blot showing claudin-5 (Cldn5) expression in hCMEC/D3-WT (7 days after seeding), hCMEC/D3-*Cldn5*-YFP (7 and 21 days after seeding) and pBCEC cultures (7 days after seeding). β-actin was used as a loading control. **g** Quantification of Western blot bands and normalization of claudin-5 expression to β-actin. Data are represented as mean ± SEM of n = 3 independent experiments. Significant intergroup differences are indicated by asterisk (*P < 0.05). **h** Representative Western blot showing Pgp expression in hCMEC/D3-WT, hCMEC/D3-*Cldn5*-YFP and pBCEC cultures. β-actin was used as a loading control. **i** Quantification of Western blot bands and normalization of Pgp expression to β-actin. Data are represented as mean ± SEM of n = 3 independent experiments. Significant intergroup differences are indicated by asterisk (*P < 0.05)

Cells were grown on filter inserts until 7 days after seeding and localization of claudin-5 was examined by immunofluorescent staining and laser scanning microscopy (Fig. 1). As shown in the confocal fluorescent microscopic images of cells (fixed with 4% paraformaldehyde) in Fig. 2a, b, claudin-5 was expressed at the cell borders of hCMEC/D3-WT cells, hCMEC/D3-*Cldn5*-YFP and pBCECs, indicating junctional localization. However, as shown by Western blot experiments, expression of endogenous claudin-5 in hCMEC/D3-WT cells was significantly lower than expression of claudin-5 in primary cultured pBCECs (Fig. 2f, g). Transfection of hCMEC/D3 cells with *Cldn5*-YFP led to a significant about sixfold increase in expression of claudin-5 (Fig. 2f, g), resulting in claudin-5-YFP levels that were not significantly different from claudin-5 levels in pBCECs (P = 0.1435). Interestingly, no endogenous claudin-5 was observed in the *Cldn5*-YFP transduced hCMEC/D3 cells. Down-regulation of endogenous mRNA and protein expression in response to transfection with an exogenous construct is a frequently observed phenomenon in cell lines, suggesting feedback-regulation of the endogenous gene to fulfil cellular needs [28].

### Morphology and Pgp expression of hCMEC/D3 cells vs. pBCECs

As shown in Fig. 2c, both hCMEC/D3-WT cells and pBCECs formed monolayers and exhibited the typical spindle-shaped morphology of BCECs when examined at confluence by phase-contrast microscopy 1–2 days post-confluency, substantiating a previous report [29]. Purity of the pBCEC isolation was evaluated by fluorescent staining for the endothelial cell marker CD31 (green in Fig. 2d), demonstrating a high purity of the culture for endothelial cells. As reported recently [29], the size of the hCMEC/D3 and porcine BCECs did not differ (Fig. 2e). Cell size was measured in ≥ 25 xy scans of cell cultures and dimensions were measured in ≥ 40 cells, respectively. Morphology of the cells was not affected by transfection with *Cldn5*-YFP (Fig. 2c). However, the Pgp expression of hCMEC/D3 cells was only about 10% that of pBCECs, which was not affected by transfection with *Cldn5*-YFP (Fig. 2h, i). Despite the relatively low expression of Pgp in hCMEC/D3 cells, which has been reported previously [8, 12, 15, 30], we and others have shown that Pgp in these cells is functional when studying Pgp substrates and inhibitors in cellular uptake experiments [8, 12, 15, 18, 31], but we repeated such experiments to demonstrate the functionality of Pgp in both WT and *Cldn5*-YFP transduced hCMEC/D3 cells (see below). Furthermore, we and others have shown previously that Pgp is located at the apical membrane of hCMEC/D3 cells [12, 18]. With respect to Pgp in pBCECs, these cells were 5 days in culture at time of Pgp determination, at which Pgp expression is ~ 70% lower than in freshly isolated pBCECs [﻿32]. The high values of Pgp expression in primary cultured pBCECs are similar to Pgp expression values in freshly prepared human brain capillaries [8].

### Transfection of hCMEC/D3 cells with Cldn5-YFP increases TEER and decreases paracellular permeability

**Fig. 3:**
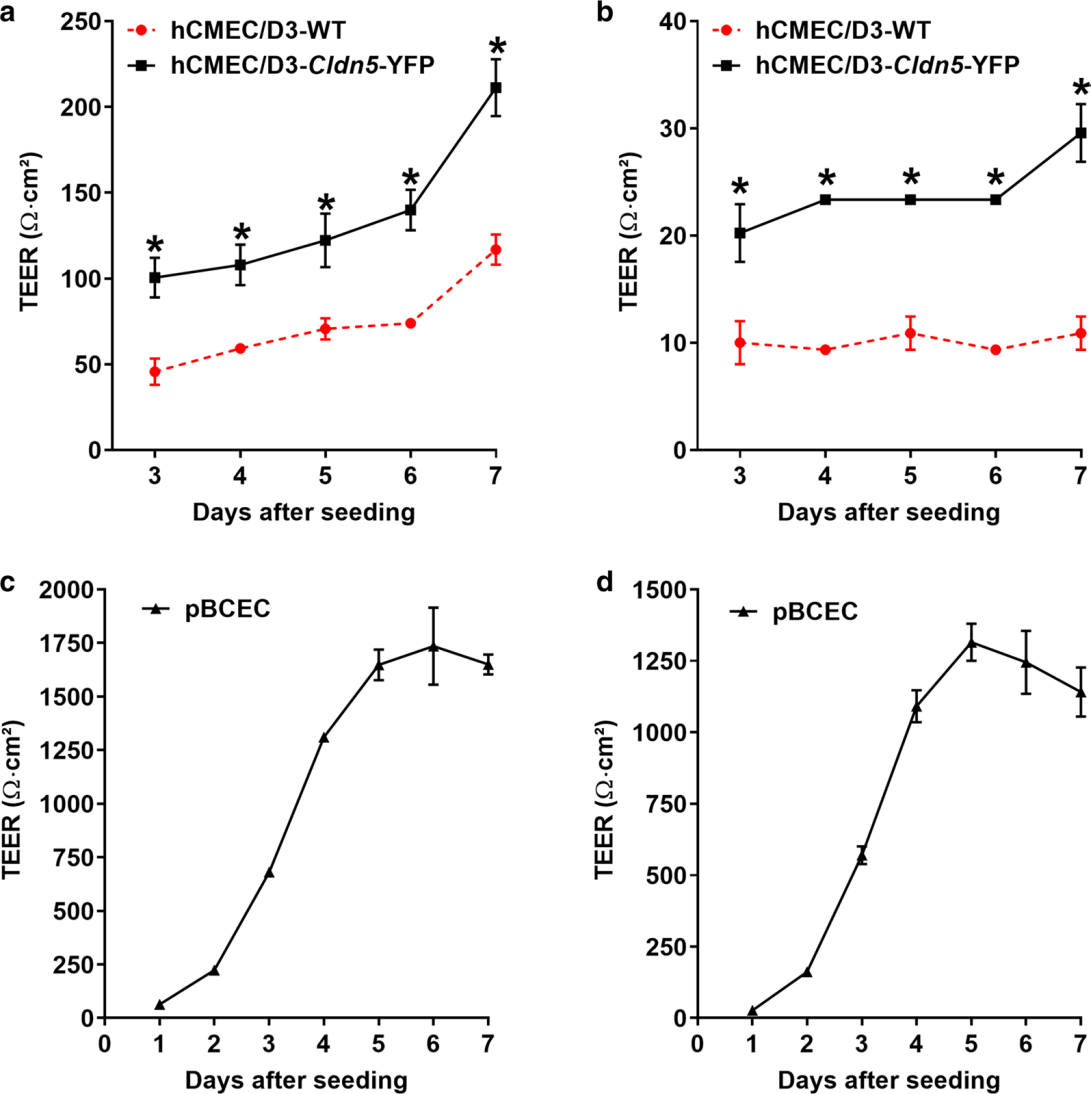
Transendothelial electrical resistance in hCMEC/D3-WT, hCMEC/D3-*Cldn5*-YFP and pBCECs. TEER values are shown as Ω cm^2^ and results are expressed as mean ± SEM of 3 replicates. Values in **a** and **c** were determined with chopstick electrodes, whereas values in **b** and **d** were determined with an EndOhm chamber. *P < 0.0001 as determined by two-way ANOVA using Bonferroni posthoc analysis

TEER reflects the ionic conductance of the paracellular pathway in an epithelial or endothelial monolayer [33]. For the present experiments, cells were grown on filter inserts and TEER was measured daily starting from day 3 (hCMEC/D3) or day 1 (pBCEC) after seeding, respectively, using an EVOM Volt-Ohm meter device and chopstick electrodes. Furthermore, for comparison, TEER values were also measured with an EndOhm chamber, as recently used by Weksler et al. [15] for hCMEC/D3 cells. Transfection of hCMEC/D3 cells with *Cldn5*-YFP significantly increased TEER compared to hCMEC/D3-WT cells (Fig. 3a, b). TEER values determined with chopstick electrodes (Fig. 3a) were higher than values determined with an EndOhm chamber (Fig. 3b), but the relative difference in TEER values between hCMEC/D3-WT and hCMEC/D3-*Cld5*-YFP cells was the same. In both cell lines TEER increased as a function of days after seeding (day 3 after seeding was equal to day 1 of confluency), presumably due to TJ maturation with time as suggested by Weksler et al. [12]. However, while TEER values in hCMEC/D3 cells still tended to increase between days 5 and 7 after seeding (Fig. 3a, b), TEER values in pBCECs reached a plateau after 5 days (Fig. 3c, d). We therefore repeated the experiment with hCMEC/D3 cells, extending the TEER measurements to 9 days after seeding. As shown in Table 1, a plateau of TEER values was reached between 7 and 9 days, thus demonstrating that a maximum of TJ formation was obtained after 7 days.

**Table 1:** Comparison of transendothelial electrical resistance (TEER) in hCMEC/D3-WT vs. hCMEC/D3-*Cldn5*-YFP cells

Day after seeding	TEER (Ω cm^2^)	
	hCMEC/D3-WT	hCMEC/D3-*Cldn5*-YFP
1	10.1 ± 0.8	15.2 ± 1.2*
2	14.8 ± 0.8	22.6 ± 0.8*
3	18.7 ± 0	26.5 ± 1.5*
4	19.5 ± 0.8	26.5 ± 1.5*
5	18.7 ± 0	26.5 ± 1.5*
6	16.3 ± 2.3	24.9 ± 1.5*
7	15.5 ± 1.5	26.5 ± 1.5*
8	16.3 ± 2.3	28.0 ± 0*
9	17.9 ± 2.1	26.5 ± 1.5*

**Fig. 4:**
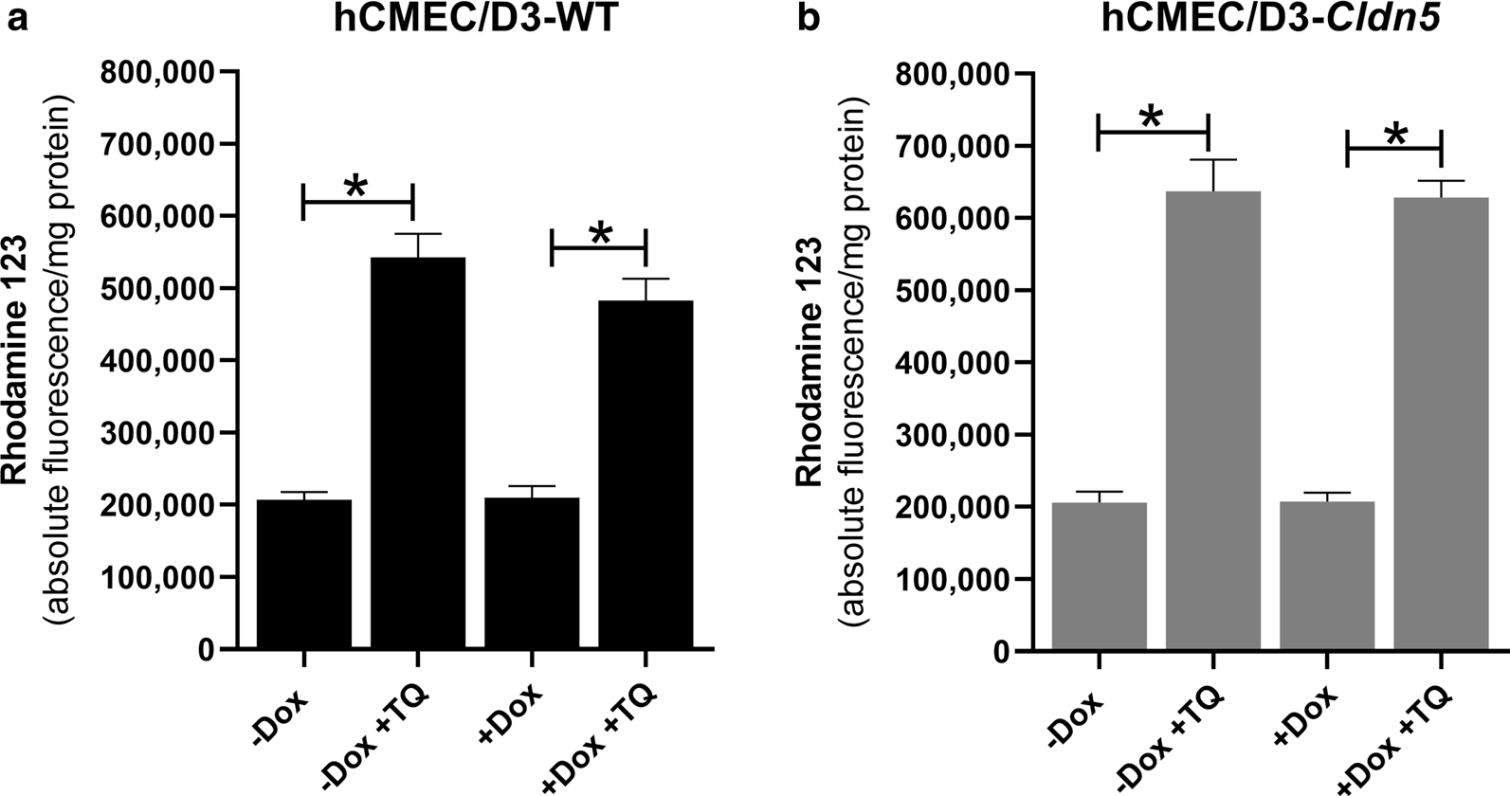
Pgp is functional in hCMEC/D3 cells as indicated by the effect of the Pgp inhibitor tariquidar (TQ; 0.5 µM) in the Rho123 uptake assay, in which alterations in Pgp efflux are indirectly measured by determining intracellular concentrations of the Pgp substrate Rho123. Data are shown as mean ± SEM of six experiments. Significant differences between treatments are indicated by asterisk (P < 0.0001). **a** Shows data from the Rho123 uptake assay in nontransduced (WT) hCMEC/D3 cells in the absence or presence of doxycycline (Dox). Doxycycline (1 µg/mL) did not alter the functionality of Pgp. Tariquidar significantly increased the uptake of Rho123 in WT cells both in the absence and presence of doxycycline to the same extent. **b** Shows data from the Rho123 uptake assay in transduced hCMEC/D3-Cldn5-YFP cells in the absence or presence of Dox. Again, Dox (1 µg/mL) did not alter the functionality of Pgp. Tariquidar significantly increased the uptake of Rho123 in the transduced cells both in the absence and presence of Dox to the same extent. Consistent with the similar expression of Pgp in the two cell lines (see Fig. 2i), the functionality of Pgp was comparable in hCMEC/D3-WT and hCMEC/D3-Cldn5-YFP cells

Data are presented as the mean ± SEM of 3 replicates. TEER was measured utilizing an EndOhm-6 tissue resistance measurement chamber. Significant difference between hCMEC/D3-WT and hCMEC/D3-*Cldn5*-YFP cells is indicated by asterisk (P < 0.05). This experiment is a repeat of the experiment shown in Fig. 4b, but TEER measurements were performed up to 9 days after seeding

When TEER data were measured with chopstick electrodes, average TEER values at 7 days after seeding were 211 Ω cm^2^ for hCMEC/D3-*Cld5*-YFP vs. 117 Ω cm^2^ for hCMEC/D3-WT cells (P = 0.0014; Table 2). Thus, transfection with *Cldn5*-YFP almost doubled TEER of the hCMEC/D3 cells. Respective values measured with an EndOhm chamber were 10.9 and 29.6 Ω cm^2^ (P < 0.01; Table 2). However, for comparison, average TEER values of 1650 Ω cm^2^ (chopstick electrodes) and 1141 Ω cm^2^ (EndOhm chamber) were determined in the primary cultured pBCECs at 7 days after seeding (Fig. 3c, d; Table 2), substantiating a marked TEER difference between cell lines and primary cultured BCECs (P < 0.001). The high TEER values in primary cultured pBCECs are in the range of well above 1000 Ω cm^2^ that is widely accepted as a characteristic of the mammalian BBB in vivo [12].

**Table 2:** Comparison of transendothelial electrical resistance (TEER) and paracellular mannitol permeability in hCMEC/D3-WT, hCMEC/D3-*Cldn5*-YFP and pBCECs

	TEER (Ω cm^2^)		Mannitol permeability	
	Chopstick	EndOhm-6 chamber	nm/s	%/h
hCMEC/D3-WT	117 ± 9	10.9 ± 1.6	111 ± 5	14.2 ± 0.4
hCMEC/D3-*Cldn5*-YFP	211 ± 8*	29.6 ± 1.6*	80.5 ± 0.7*	8.06 ± 0.1*
pBCEC	1650 ± 46^#^	1141 ± 86^#^	23.3 ± 5^#^	3.95 ± 0.6^#^

Data are presented as the mean ± SEM of 3 replicates. For TEER, which was measured by either chopstick electrodes or an EndOhm-6 tissue resistance measurement chamber, significant difference between hCMEC/D3-WT and hCMEC/D3-*Cldn5*-YFP cells is indicated by asterisk (P < 0.01), whereas significant difference between pBCECs versus hCMEC/D3-WT and hCMEC/D3-*Cldn5*-YFP cells is indicated by the hash sign (P < 0.001). For mannitol values, significant difference between hCMEC/D3-WT and hCMEC/D3-*Cldn5*-YFP cells is indicated by asterisk (P = 0.0040), whereas significant difference between pBCECs versus hCMEC/D3-WT and hCMEC/D3-*Cldn5*-YFP cells is indicated by the hash sign (P < 0.001)

Restriction of paracellular molecular flux of ions and other small hydrophilic solutes is one of the most important characteristics of TJs [6, 7]. To trace paracellular flux to small hydrophilic solutes here, we used the monosaccharide mannitol (molecular weight, 182 Da) because it is not subject to active transcellular transport and widely used as a paracellular flux marker [34]. [^14^C]-mannitol flux across the cellular monolayer was assessed at day 7 after seeding in basal to apical (b-A) direction as paracellular permeability marker. Table 2 shows TEER values of the 2 cell lines and pBCECs at day 7 after seeding as well as mannitol permeability across the monolayer in nm/s and percent per hour.

It is well known that TEER is negatively correlated with paracellular permeability of polarized endothelial and epithelial cells, i.e., the higher the TEER the lower the paracellular permeability to ions and small hydrophilic solutes such as mannitol, which is a consequence of TJ protein expression [33, 35, 36]. Such a correlation was also obtained in the present experiments. As shown in Table 2, hCMEC/D3-WT cells had a high paracellular permeability (indicating paracellular leakage through endothelial TJs) with an average mannitol flux value of 111 nm/s or 14% per h. Paracellular permeability was significantly reduced by transfection of hCMEC/D3 cells with *Cldn5*-YFP, resulting in an average mannitol flux value of 81 nm/s or 8% per h. However, this was still high above the low mannitol flux values determined in pBCEC (23.3 nm/s or 4%/h).

### Pgp is functional in hCMEC/D3-WT and hCMEC/D3-Cldn5-YFP cells when using a rhodamine 123 uptake assay

As shown in Fig. 4, in both hCMEC/D3-WT and hCMEC/D3-*Cldn5*-YFP cells, Pgp was functional, because the accumulation of Rho123 in the cells was significantly increased by the Pgp inhibitor tariquidar. Doxycycline did not alter the functionality of Pgp in the cells. Consistent with the similar expression of Pgp in the two cell lines (see Fig. 2i), the functionality of Pgp was comparable in hCMEC/D3-WT and hCMEC/D3-*Cldn5*-YFP cells (Fig. 4a, b).

### In contrast to pBCECs, neither hCMEC/D3-WT nor hCMEC/D3-Cldn5-YFP cells allow bidirectional (vectorial) drug transport studies in Transwells

**Fig. 5:**
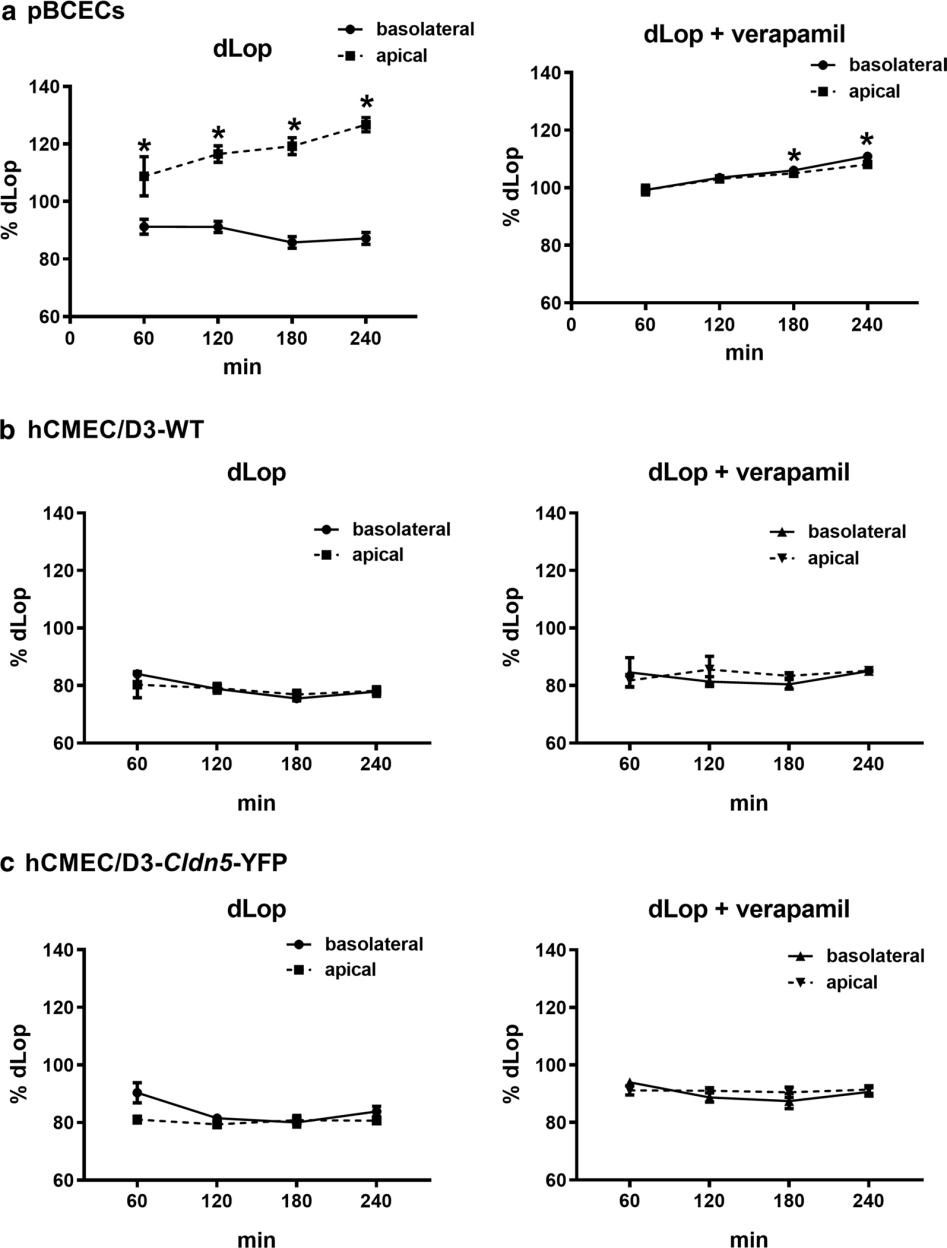
Concentration equilibrium transport assay (CETA) of Pgp substrate *N*-desmethyl-loperamide (dLop) in hCMEC/D3-WT, hCMEC/D3-*Cldn5*-YFP and pBCECs. Data are shown as percentage of the initial drug concentration (= 100%) in the apical and basolateral chamber of the Thincert system over time. **a** An increase of the drug concentration in the apical chamber and a simultaneous reduction in the basolateral chamber indicates drug transport by Pgp across the primary pBCEC monolayer. Almost no drug transport was measurable when the experiment was repeated in the presence of the Pgp inhibitor verapamil (50 µM) as a control. **b**, **c** A Pgp-mediated ([^3^H]dLop) transport across the hCMEC/D3 monolayer could not be observed

For drug transport studies, cells were seeded on inserts and CETA was performed 7 days after seeding. Radiolabeled [^3^H]*N*-desmethyl-loperamide ([^3^H]dLop) was added to both (apical and basolateral chamber) sides of the monolayer and concentrations were measured in both chambers over 240 min. As shown in Fig. 5a, significant Pgp-mediated transport of the selective Pgp substrate dLop [37] was determined in pBCECs. As shown previously for various other Pgp substrates in the CETA assay [27, 38–42], asymmetrical (basolateral to apical) transport of dLop was indicated by the significant increase of drug concentration in the apical chamber and simultaneous decrease of drug concentration in the basolateral chamber, demonstrating basolateral-to-apical drug transport across the pBCEC monolayer in the Transwells. This transport was almost completely inhibited by the Pgp inhibitor verapamil, substantiating our previous data [25] that the bias of passive drug diffusion between chambers is minimized in the CETA assay.

In contrast to active basolateral-to-apical transport of dLop by pBCECs, no such transport was determined when using hCMEC/D3-WT or hCMEC/D3-*Cldn5*-YFP cells (Fig. 5b, c). The most likely explanation for the lack of any significant asymmetrical transport of dLop in the CETA assay when using hCMEC/D3 cells is the high paracellular permeability of these cells determined by the mannitol experiments shown in Table 2, because high paracellular permeability would lead to rapid exchange of small molecules between both Transwell chambers, thus masking any directed (vectorial) drug transport from basolateral to apical chamber [8].

## Discussion

Transduction of hCMEC/D3 cells with claudin-5 resulted in an about sixfold increase in the expression of the TJ protein, so that expression levels of claudin-5 were in the range of those determined in primary cultured pBCECs. As expected, the claudin-5 transduced hCMEC/D3 cells exhibited significantly increased TEER values and reduced paracellular permeability compared to hCMEC/D3-WT cells. However, despite the similar claudin-5 expression of hCMEC/D3-*Cldn5*-YFP and pBCECs, the primary cultured porcine cells had much higher TEER values and significantly lower mannitol permeability than the claudin-5 transduced hCMEC/D3 cells, indicating that claudin-5 alone is not responsible for high TEER and low paracellular permeability values of BCECs. Most likely as a consequence of the relatively low tightness of hCMEC/D3 monolayers, no vectorial transport of a lipophilic small molecule Pgp substrate (dLop) was observed, irrespective of whether WT or claudin-5 transduced cells were used. In contrast, such drug transport could be easily demonstrated by using primary cultured pBCECs.

The high TEER values determined in monocultures of pBCECs are in line with values reported in the literature [8] and almost reach values of the in vivo BBB (usually in the range of 1800 to 2000 Ω cm^2^) [10]. Literature TEER data for monocultures of hCMEC/D3 cells range between 8 and 300 Ω cm^2^ [8, 12] depending on differences in measuring equipment and on whether hydrocortisone was added to the culture medium to reinforce BBB properties as done in the present study. TEER obtained by separate groups in separate studies may differ somewhat, not only because of differences in actual junctional tightness but also because of differences in measuring equipment [8]. As shown here by directly comparing two TEER measuring methods in two in vitro BBB models, data obtained with chopstick electrodes in hCMEC/D3 cells were markedly higher than data determined with an EndOhm chamber. STX2/chopstick electrodes cannot deliver a uniform current density over a relatively large membrane, which may lead to an overestimation of the TEER value, whereas in an EndOhm chamber both the chamber and the cap contain a pair of concentric electrodes, thus allowing for a more uniform current density across the membrane [33].

To our knowledge, only one previous study generated claudin-5 overexpressing hCMEC/D3 cells [43]. By silencing and overexpressing the *CLDN5* gene in hCMEC/D3 cells, Ma et al. [37] reported that the claudin-5 overexpressing cells exhibit a decreased paracellular permeability to cancer cells. However, TEER or permeability to small paracellular permeability markers (such as sucrose or mannitol) were not examined. It is well known that the hCMEC/D3 BBB model in its basic state creates a barrier for large molecules, whereas small molecules can easily penetrate through the barrier [8]. As shown here, this is not changed by overexpressing claudin-5.

Various previous studies have used diverse manipulations to increase TEER in hCMEC/D3 cells, including coculturing with astrocytes or pericytes, exposure to hydrocortisone (which was also used in the present study), simvastatin or physical (pulsatile flow-induced) shear stress, addition of human serum, activating the Wnt/β-catenin pathway, the Wnt/planar cell polarity pathway or nuclear receptors [8, 12]. Although all these manipulations increased TEER by different extent, it has not been demonstrated convincingly that the junctional tightness of the hCMEC/D3 model to small molecules is sufficiently improved to allow vectorial drug transport studies.

In a series of previous experiments of the use of hCMEC/D3 cells for drug transport studies [44], we studied TEER, paracellular mannitol permeability, and permeability to Pgp substrates of hCMEC/D3-WT cells in the Transwell system with and without astrocyte coculturing or exposure to hydrocortisone or simvastatin, or addition of human serum as proposed by Poller et al. [45] Moreover, the influence of different culture media and membrane materials in the Transwells was evaluated. In these experiments, we used different small molecule Pgp substrates, including Rho123 and digoxin, and different Pgp inhibitors, including PSC833 (valspodar) and tariquidar. Furthermore, CETA and traditional bidirectional transport assays, in which the drug is only added to one (e.g., the basolateral) chamber of the Transwell system, were compared. Although several manipulations increased TEER and decreased paracellular mannitol flux of the hCMEC/D3 cell monolayers, in none of these numerous experiments, any robust asymmetrical transport of Pgp substrates was observed [44]. In contrast, significant asymmetrical (basolateral to apical) transport of known Pgp substrates was easily determined when we used kidney epithelial cells (*ABCB1*-transfected MDCK-II and LLC-PK1 cells) as surrogate for BCECs in the Transwell assay [27, 39, 44, 46, 47].

In our previous studies in MDCK-II and LLC-PK1 cells, only monolayers with a TEER of at least 100 Ω cm^2^ were used for analysis of drug transport, which was previously recommended in the FDA guidance for such studies [25]. Furthermore, with respect to paracellular permeability of the cell monolayers, we used < 1% of mannitol diffusion per hour, and an apparent permeability (Papp) of [^14^C]-mannitol < 12 nm/s as indicators of monolayer integrity [27]. In the FDA guidance for such studies, mannitol permeability values of 2–20 nm/s are considered sufficient to exclude paracellular permeability that would otherwise form a bias in in vitro transport studies [25]. For primary cultures of BCECs, cutoff values of 20–40 nm/s have been suggested for paracellular permeability [48]. The present mannitol permeability values determined in primary cultured pBCECs fulfilled this criterion, whereas the mannitol permeability values determined in the hCMEC/D3-WT and claudin-5 transduced hCMEC/D3 cells were significantly higher, thus explaining that these cells are not suited to study transcellular transport of small Pgp substrates.

The permeability of paracellular probes such as sucrose has been used previously to quantitate the tightness of the in vivo BBB [48]. Depending on the administration and sampling technique, sucrose BBB permeability in the rat in vivo has been determined at 0.03 to 0.1 × 10^−6^ cm/s [48]. Estimates of TEER in in vivo cerebrovascular capillaries were reported to be in excess of 1000 Ω cm^2^ [48]. Although TEER and paracellular permeability are both useful indicators of the tightness of in vitro BBB models, the measurement of TEER alone does not provide sufficient information on a restrictive paracellular pathway [48]. In this respect, the absolute permeability of the model to hydrophilic solutes such as sucrose or mannitol is more informative [2, 48–50]. Thus, the optimal characterization of paracellular permeability in an in vitro model of the BBB should include both TEER and paracellular tracer flux, as done here.

TJs and adherens junctions are the major structures that determine the paracellular permeability of the brain endothelium [51]. Among the various TJ proteins, claudin-5 is considered to play a dominant role for brain endothelial TJs [51, 52]. *Cldn5* knock-out mice display increased BBB permeability for molecules < 800 Da [53]; however, the BBB, especially the TJ morphology, appear ultrastructurally normal in these mice, suggesting that other claudins or TJ-associated marvel proteins (TAMPs) are involved. In immortalized BCEC lines from different species, claudin-5 appears to be the prominent TJ protein, because most other TJ proteins have been downregulated more extensively than claudin-5 [54]. Indeed, a recent study on tetraspanning TJ transcripts/proteins in micro dissected human and murine brain capillaries, quickly frozen to recapitulate the in vivo situation, demonstrated up to a dozen TJ proteins in brain capillaries, with high transcript expression of *CLDN5* (22% from the total), *CLDN11* (13%), *CLDN12* (8%), *CLDN25* (48%), and occludin, but also further abundant levels of *CLDN1* and *CLDN27* in humans [54]. Thus, these recent data indicate that claudin-25, and not claudin-5, is the dominant claudin isoform in the human BBB in vivo. However, in contrast to the in vivo situation, claudin-5 dominates BBB expression in vitro, since all other TJ proteins are at comparably low levels or are not expressed [54]. As a consequence, transfection of BCECs such as hCMEC/D3 with only *Cldn5*, as done here, may be insufficient to decrease paracellular permeability to levels observed in primary cultured BCECs or the BBB in vivo, which is demonstrated by the present data.

In addition to claudins, the TJ backbone in BCECs consists of other transmembrane proteins (occludin, junctional adhesion molecules [JAMs], as well as endothelial selective adhesion molecule [ESAM]), which form a complex of proteins that spans the intercellular cleft [6, 7, 55]. The transmembrane proteins recruit a number of membrane-associated cytoplasmic proteins, such as the zona occludens protein ZO-1 [6, 7]. Furthermore, VE-cadherin, an endothelial-specific transmembrane protein, plays a role in the maintenance of cell–cell junction stabilization and regulation of vascular barrier integrity [7]. All these proteins interact synergistically in determining paracellular permeability of the BBB [6, 7, 55]. Comparison of transcriptional and proteomic profiles of hCMEC/D3 cells and primary human BCECs with freshly isolated mouse BCECs confirmed the expression by hCMEC/D3 cells of a substantial number of junctional genes and proteins expressed by brain endothelium, but showed lower expression of occludin, JAM-2, and particularly ZO-1 and claudin-5 [8, 56–58]. For claudin-5, the protein expression was 5.27-fold greater in human brain microvessels than in hCMEC/D3 cells [57], which prompted the present study to transfect hCMEC/D3 cells with claudin-5.

In contrast to hCMEC/D3 cells, primary monocultures of pBCECs highly express TJ proteins such claudin-5, ZO-1 and -2, and occludin, as determined by real time PCR, Western blotting, and confocal- and electron microscopy [8]. This leads to well-differentiated TJs and high barrier tightness that closely resembles the BBB in vivo as indicated by high TEER values and low paracellular permeability as also shown here, thus providing a functional in vitro model of the BBB [8]. Furthermore, the polarized expression and functionality of the major efflux transporters, including Pgp and BCRP, in pBCECs makes this an ideal model for studying vectorial drug transport [8] as shown here for the selective Pgp substrate dLop. In a recent study in which the efflux transport of known Pgp and BCRP substrates was compared in pBCECs and an in vitro human BBB model derived from induced pluripotent stem cells (iPSCs), comparable drug permeability was obtained [59].

In a previous study, we showed that transfection of canine MDCK-II cells with a murine *Cldn5*-YFP fusion plasmid increased TEER and decreased paracellular flux of fluorescein [16], confirming previous experiments with claudin-5 transfection of MDCK-II cells [60]. Similarly, stable transfection of Caco-2 cells with FLAG-claudin-5 cDNA significantly increased TEER and reduced mannitol flux in cells with low genuine transepithelial resistance [22]. In contrast, changes of barrier properties were not detected in cells exhibiting a high TEER (MDCK-C7) following stable transfection with claudin-5 cDNA [22]. Transfection of rat BCECs with claudin-5 significantly decreased the permeability to the paracellular diffusion marker inulin [61], substantiating the role of claudin-5 in the barrier properties of brain capillary endothelial cells. However, none of these claudin-5 transfection studies demonstrated that the transfected cells can be used for examining vectorial drug transport.

Our study has a number of potential limitations. First, one could argue that the apical Pgp expression of the hCMEC/D3 cells used here is not high enough to allow studying Pgp-mediated transport by these cells. Indeed, as shown here, most likely as a consequence of cell immortalization, long-term in vitro cell expansion and culture conditions, the Pgp expression of hCMEC/D3 cells was only about 10% that of pBCECs, which was not affected by transfection with claudin-5. Similarly, Dauchy et al. [30] reported that gene and protein expression of *MDR1*/Pgp by hCMEC/D3-WT cells is markedly lower than that described for freshly prepared brain microvessels from humans. Despite the low expression of Pgp, our present and previous [31] kinetic uptake studies into cultured hCMEC/D3-WT cells, using the Rho123 efflux assay and selective Pgp inhibitors, demonstrated a clear functionality of Pgp in these cells, substantiating several other studies using this approach [12]. In subsequent studies, we transduced hCMEC/D3 cells with a doxycycline-inducible *MDR1*-EGFP fusion plasmid, which led to a markedly increased efflux of the Pgp substrate Rho123 in an efflux assay [18]. However, use of these *MDR1*-EGFP transduced cells in a Transwell assay did not allow studying vectorial drug transport because of too high paracellular permeability (unpublished data). These data are in apparent contrast to a study by Poller et al. [45], who reported vectorial transport of a Pgp substrate by using hCMEC/D3 monolayers in a Transwell system, which, however, could not be reproduced by other groups [8, 51] and the present study, because in most studies the barrier properties of the endothelial monolayer were not suitable for drug transport studies [51].

A second potential limitation is the use of monoculture systems in the present experiments. Indeed, coculture systems (with BCECs and astrocytes or pericytes) and triple culture models (with BCECs, astrocytes and pericytes) are now widely used as in vitro models of the BBB [8]. Both astrocytes and pericytes contribute to the tightness of BCEC monolayers, but the exact mechanisms of junctional regulation remain to be established [8]. However, our own experiments with cocultures of hCMEC/D3 cells and astrocytes showed only slight alterations in TEER and mannitol permeability and no significant vectorial transport of Pgp substrates [44], so that such cocultures were not used here. Similarly, Eigenmann et al. [9] and Biemans et al. [58] observed no significant increase of TEER by coculturing of hCMEC/D3 cells with astrocytes, whereas significant TEER increases were reported by Hatherell et al. [62]. Additional transfection of hCMEC/D3 cells with other TJ proteins (e.g., claudin-25 and ZO-1) and characterization of such cells in a triple culture model would be an interesting option for future experiments.

In addition, the BBB constructs of Transwell systems are still 2D planar models with less resemblance to the 3D microscale capillary architecture of the BBB in vivo [63]. With the advancement of different microscale technologies, numerous microdevices have been developed to provide in vivo-like microenvironments and 3D culture models [63]. Such microfluidic based models have many advantages over existing in vitro static models, including shear stress stimulation.

Indeed, a further potential limitation of the current approach is the lack of pulsatile flow-based shear stress, which affects barrier properties and drug transport [8]. Pulsatility is not only important in terms of shear stress, but also in terms of pressure and concentration gradients, which will affect molecule transport. In hCMEC/D3 monolayers subjected to pulsatile flow after seeding in a capillary cartridge system, the TEER was reported to rise to 1000–1200 Ω cm^2^ [64] i.e., values similar to those obtained in pBCECs. The high TEER was associated with low permeability to the paracellular permeability marker sucrose. Co-culture with astrocytes did not induce any further increases in TEER values in this flow-based model suggesting that, at least in vitro, shear stress may be a more critical factor in inducing a mature barrier phenotype than interactions with other cell types.

## Conclusions

As outlined by Helms et al. [8], the hCMEC/D3 BCEC line provides an easy to use, extensively characterized in vitro model of the human BBB, which is well suited for drug uptake studies and for examining the response of brain endothelium to human pathogens and neuro inflammatory processes. However, the low junctional tightness of hCMEC/D3 cells excludes the use of this BBB model for bidirectional (vectorial) transport studies on small compounds such as the Pgp substrate dLop used here. As shown here, this problem cannot be resolved by transfection with claudin-5, i.e., the dominating TJ protein in the hCMEC/D3 cell line. In this respect, more recent BBB models generated from human iPSCs are advantageous, providing high TEER and low paracellular permeability similar to those of primary cultured BCECs [65–67]. However, one should consider that the differentiation capacities of human iPSCs vary depending on the initial source of cells. Furthermore, detailed information on the long-term stability of these cells is not available, which may affect drug screening [63]. Thus, as in other areas of biomedicine, there is no ideal in vitro model of the BBB, but the appropriate model has to be selected with respect to the information expected to be obtained from the study, using fit-for-purpose principles [68, 69]. The hCMEC/D3-*Cldn5*-YFP cells presented here provide an interesting model for studying the contribution of claudin-5 to barrier tightness and how this can be further enhanced by additional transfections or other manipulations of this widely used in vitro model of the BBB.

## Data Availability

The datasets used and/or analyzed during the current study are available from The corresponding author on reasonable request.

## Abbreviations

BBB: Blood–brain barrier
BCEC: Brain capillary endothelial cell
BCRP: Breast cancer resistance protein
CETA: Concentration equilibrium transport assay
*Cldn5*: Claudin-5
dLop: Desmethyl-loperamide
FCS: Fetal calf serum
Pgp: P-glycoprotein
Rho123: Rhodamine 123
TEER: Transendothelial electrical resistance
TJ: Tight junction
WT: Wildtype
YFP: Yellow fluorescent protein
